# A Comparative Study of Two WAAM Patterns for Structures with Grid Fin Characteristics

**DOI:** 10.3390/ma18020219

**Published:** 2025-01-07

**Authors:** Guo Xian, Jingbang Pan, Qingshan Li

**Affiliations:** 1 Key Laboratory of Industrial Control Technology, Institute of Cyber-Systems and Control, Zhejiang University, Hangzhou 310058, China; 2 Huzhou Institute, Zhejiang University, Huzhou 313000, China; 3 Department of Architecture and Civil Engineering, University of Bath, Bath BA2 7AY, UK; 4 Zhejiang Academy of Special Equipment Science, Hangzhou 310009, China; liqs@zjtj.org

**Keywords:** grid fin, WAAM pattern, printability, microstructure, residual stress

## Abstract

As a critical component of rocket systems, the grid fin is widely applied in the aerospace industry. Compared to traditional manufacturing methods and other additive manufacturing (AM) techniques, wire arc additive manufacturing (WAAM) is more advantageous in its time and cost efficiency, especially when it is utilized in the large-scale production of components. Given the significant effect of the welding strategies on the quality of manufactured parts, we investigated two distinct WAAM printing orientations, horizontal, or lie (L), and perpendicular, or stand (S), using a small-scale model. The feasibility of these two printing approaches was evaluated by analyzing the surface quality, microstructure, and mechanical properties at the junctions of the grid fin. Furthermore, a finite element analysis (FEA) was adopted to simulate and analyze the main factors, including the temperature distribution, deformation, and stress profiles at the welding joints, in both AM strategies. The integrated approach adopted in this study provides important insights for optimizing the application of WAAM in grid fin manufacturing. In summary, our results indicate that while the L mode is easily manufacturable and exhibits stable properties, the S mode holds significant market potential if its welding parameters are optimized.

## 1. Introduction

Aerospace, an important industry to national strategic development, has profound implications for both defence security and technological advancement [1]. Breakthroughs in critical technologies, such as the development of rockets, aircrafts, and missiles, have revolutionized the defence capabilities and global competitiveness of various nations. Among these, rockets facilitate space exploration, highlighting the need to develop advanced space technologies. However, rocket manufacturing is an intricate and resource-intensive process. Components such as engines, fuel systems, and key structural parts require exceptional precision and durability. Moreover, advanced manufacturing techniques, such as additive manufacturing (AM), are utilized to reduce costs and improve the efficiency in material use and fabrication [2].

The grid fin, assembled at the tail of rockets, is a critical component involved in flight control [3]. It allows for control of a rocket’s attitude and trajectory owing to its grid-like structure. However, its complex geometry creates challenges for the traditional manufacturing techniques, particularly for large-scale components exceeding three meters in size. This is because the traditional methods do not effectively balance the requirements for a grid fin to be lightweight and high-strength under complex stress conditions. Furthermore, as the dimensions of a small-scale end-use part increases, it becomes increasingly challenging to cut and machine the material, which causes significant material waste and high production costs and enhances the fabrication time. These limitations underscore the need for the adoption of advanced manufacturing technologies, such as AM, to improve the efficiency and reduce costs. In practice, 17-4PH stainless steel is used due to its low costs and strong mechanical properties compared to other materials. To compensate for its higher density and weight, WAAM technology is adopted to obtain a lightweight design.

WAAM is a novel technology belonging to the Directed Energy Deposition (DED) category of AM which is increasingly applied in the aerospace industry. Notably, WAAM can build components layer by layer using welding wire as the filler material and an arc as the heat source [4]. WAAM is also an environmentally friendly manufacturing technique that is safe and cost-effective. Unlike traditional AM techniques, which are constrained by the limited working spaces of their equipment, WAAM provides unparalleled flexibility in terms of the equipment and operational range, making it ideal for fabricating oversized components [5,6]. It has several advantages, including minimal spatial constraints in large-scale printing, making it broadly applicable across industries such as aerospace, marine engineering, and heavy machinery. To date, WAAM has been successfully applied in the fabrication of large rocket components, aircraft wings, and ship hulls, suggesting that it has great potential for grid fin manufacturing [7].

The manufacture of a grid fin component using WAAM is achieved through two approaches: horizontal and vertical. Currently, horizontal printing is the predominant AM approach for complex grid structures [4]. In this “lie” mode, the grid fin lies horizontally, while the layers are printed in the vertical direction. This method is widely applied in Powder Bed Fusion (PBF) and DED laser printing and is particularly suitable for medium and small experimental samples. So far, previous studies have focused on the feasibility of small-scale grid intersections, evaluating their surface quality, structural performance, and printing feasibility. Although such investigations have generated important insights for exploring grid structures in AM, they have been limited to small samples, and little knowledge regarding large-scale grid fins is available [8,9]. Some companies, such as India’s Ankit Aerospace, have utilized Electron Beam Additive Manufacturing (EBAM) to fabricate grid fins, such as for the Gagan Yaan mission. Similarities have also been drawn to SpaceX-style grid fins; however, detailed descriptions of the production process and experimental data have not been given to guide engineering implementations. Perpendicular printing, or the “stand” mode, is considered an effective alternative AM approach in which the grid structures are constructed layer by layer in the vertical direction. Theoretically, this method appears to be ideal for grid fins because their horizontal dimensions are significantly larger than their vertical ones. Vertical printing enables the production of oversized components on smaller platforms, negating the need for larger equipment. However, despite these theoretical advantages, vertical printing faces challenges such as heat input control, material deformation, cracking prevention, and path optimization. Consequently, few studies have been conducted to explore its application in large-scale grid structures.

This study addresses the challenges and technical constraints associated with grid fin manufacturing [10]. Detailed experiments were conducted in two WAAM printing orientations: “lie” (L) and “stand” (S). In the study, we balanced these factors while considering cost, manufacturing time, structural integrity, and other relevant aspects. A comprehensive comparative analysis was performed to determine its feasibility for additive manufacturing and the surface quality, microstructure, and mechanical properties at the joints [11,12,13,14]. The thermal history and residual stress were analyzed under the two deposition modes. This study provides key insights for optimizing printing solutions under various conditions, highlighting the associated risks and challenges, and contributing to the advancement of grid fin manufacturing.

## 2. Experimentation

### 2.1. Materials and the WAAM Process

**Figure 1:**
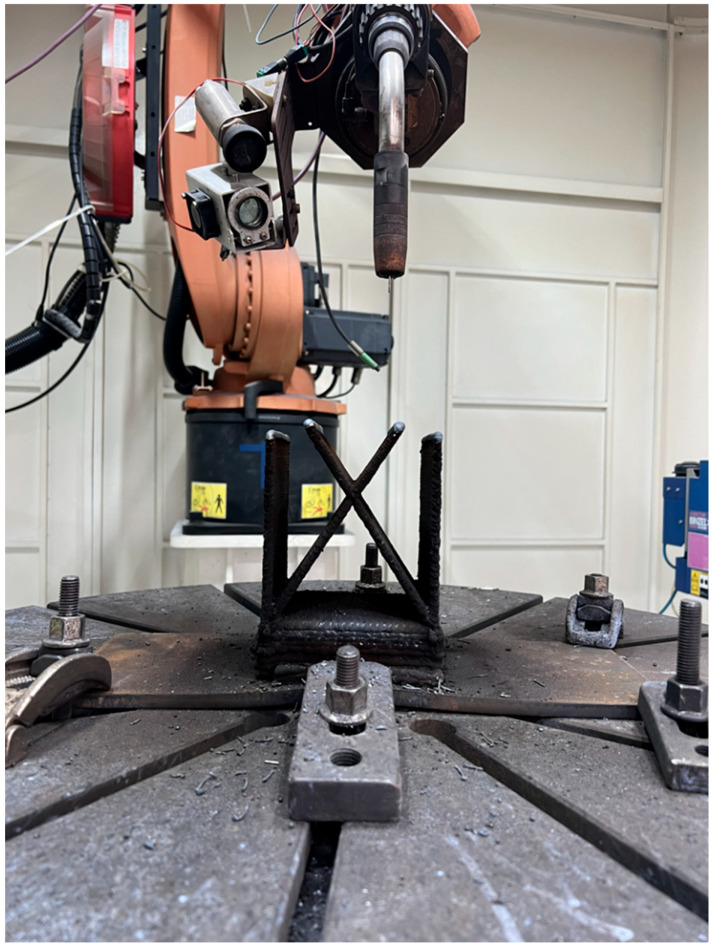
An illustration of the wire arc additive manufacturing setup for small-scale grid fin building.

Briefly, the WAAM specimens were fabricated using CMT and a 17-4PH stainless steel consumable with a diameter of 1.2 mm; its chemical composition is illustrated in Table 1. Q235 carbon steel fixed onto the work platform was used as the base metal, as shown in Figure 1. Table 2 shows the processing parameters applied in the experiment. The thickness of a single wall was maintained at about 4 mm. The robot was coupled with a camera to facilitate measurement and monitoring of the highest weld metal temperature, thus ensuring a constant interpass temperature. We chose 98% Ar + 2% CO_2_ as the shielding gas instead of 99.99% Ar to the minimize production costs. This was due to the observation that both could produce the same morphology and properties in trial–error experiments. Single-wall deposition was carried out, and intersection joint structures of different thicknesses were also manufactured to fabricate the grid fin structure through two approaches, including under the “stand” (S) mode. Studies have demonstrated that the “lie” (L) mode is a more practical approach. In current practical applications, the oscillation strategy is often utilized to design thick grid fins instead of multi-pass for a single layer, with different angles (ranging from 45 to 55 degrees) also being adopted to test the limitations of the WAAM technology in the absence of a support. In all of the tests, a small-scale component was fabricated under the “lie” (L) and “stand” (S) modes.

### 2.2. Characterization

**Figure 2:**
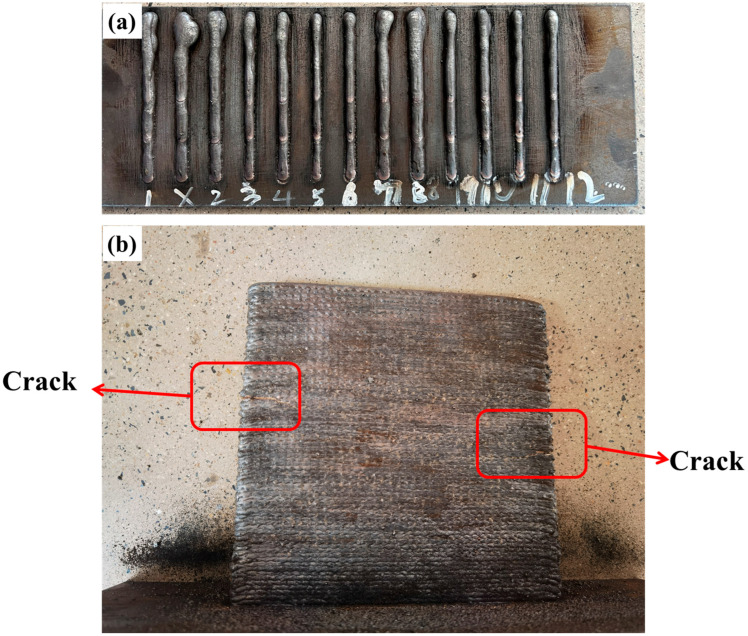
Illustration of welding parameter optimization (**a**) and defects in single-wall deposition (**b**).

**Figure 3:**
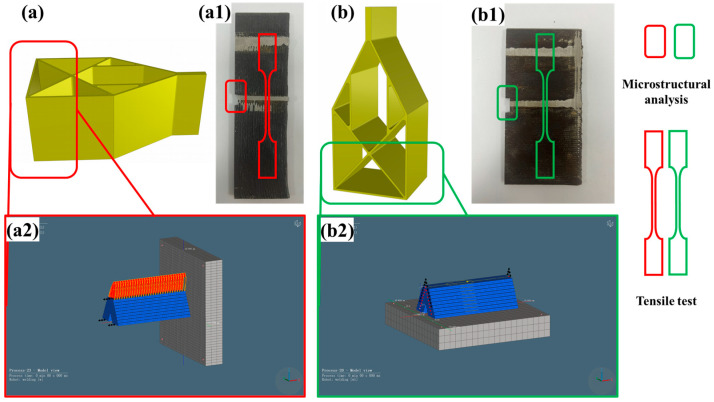
Geometry structure, mesh construction, and intersection joint test: (**a**,**a1**,**a2**) correspond to the L mode, and (**b**,**b1**,**b2**) correspond to the S mode.

The optimal parameters for the process were explored through multiple optimization tests and adjustments. More than 12 welding parameters (Figure 2a) were tested to determine the optimal conditions for achieving a better weld pool quality. If the welding parameters were not ideal, defects such as cracks (Figure 2b) formed on the sides of the single-wall structure. After deposition of the single-wall sample, a Keyence digital microscope (VHX-7000, Osaka, Japan) was employed to perform a 3D imaging analysis of the wall’s surface, which allowed for a comprehensive analysis of its surface quality. In this analysis, we characterized the surface morphology and roughness, ensuring the compliance of the surface characteristics of the prepared samples with the design requirements. Further characterization was conducted on the intersection joint under two modes (L and S). Initially, a Stereo Microscope (ZEISS, Oberkochen, Germany) was utilized to examine the macroscopic morphology, and different printing modes were employed to observe the defects and quality of the junctions assessed. Subsequently, the microstructure, crystallinity, and phase distribution were examined using an Optical Microscope (OM, Olympus BX51M, Tokyo, Japan), a Scanning Electron Microscope (SEM, Zeiss, Gemini 500; Oberkochen, Germany), and Electron Backscatter Diffraction (EBSD, AMETEK EDAX; 15 kV; Harrisburg, PA, USA). The mechanical properties at the junctions were determined using a hardness test (see details in Figure 3(a1,b1)).

### 2.3. Thermal History and Stress Simulation of WAAM Deposits

To establish a thermal model, we employed the finite element method and the Simufact Welding 2022 software to characterize the microstructural evolution and reliability of the components. Of note, we could not make calculations for all of the components under the limits on computation time and space owing to their large size and complex structure. The adoption of simplified structural modeling minimized the costs, predicted the trends in the defects during the WAAM process, and shortened the welding process time. The mesh’s construction was simplified and modified to obtain results rapidly and efficiently, with emphasis on the process with the two different printing modes (L and S) at the intersection (details shown in Figure 3(a2,b2)). The deposition width was 5 mm, while the height was 1 mm. The modeling mesh contained a hexahedral structure (0.5 mm × 0.5 mm × 0.5 mm), which was more stable, and a heat source model, which is often employed in arc welding, with a double-ellipsoidal shape. The differential equation utilized in the transient thermal analysis is expressed as follows [15]:

(1) $$\rho c_{p}\frac{\partial T}{\partial t}=\frac{\partial }{\partial x}\left(k_{x}\frac{\partial T}{\partial x}\right)+\frac{\partial }{\partial y}\left(k_{y}\frac{\partial T}{\partial y}\right)+\frac{\partial }{\partial z}\left(k_{z}\frac{\partial T}{\partial z}\right)+\dot{q}$$

where ρ indicates the density of the conducting medium; $c_{p}$ denotes the specific heat of the medium; $k_{x}$, $k_{y}$, $k_{z}$ represent the thermal conductivities of the medium in the x, y, and z directions, respectively; t indicates time; and $\dot{q}$ denotes the total heat input based on Goldak’s double-ellipsoidal model. This differential equation was solved under the following assumptions.

(1) The initial temperature of the model is initially 20 °C;
(2) The fluid flow in the molten pool is negligible;
(3) The heat transfer from the model to the environment is defined at the model’s surface as convective heat dissipation and radiation to the environment at 20 °C.

The power densities of the double-ellipsoid heat source during the WAAM process, denoted as $q_{f}\left(x,y,z\right)$ and $q_{r}\left(x,y,z\right)$ and used to describe the heat flux distributions in the front and rear quadrants, respectively, are expressed as Equations (2) and (3):

(2) $$q_{f}\left(x,y,z\right)=\frac{6\sqrt{3}f_{f}Q}{a_{f}bc\pi \sqrt{\pi }}\cdot e^{-\frac{3x^{2}}{a_{f}^{2}}}\cdot e^{-\frac{3y^{2}}{b^{2}}}\cdot e^{-\frac{3z^{2}}{c^{2}}}$$

(3) $$q_{r}\left(x,y,z\right)=\frac{6\sqrt{3}f_{r}Q}{a_{r}bc\pi \sqrt{\pi }}\cdot e^{-\frac{3x^{2}}{a_{r}^{2}}}\cdot e^{-\frac{3y^{2}}{b^{2}}}\cdot e^{-\frac{3z^{2}}{c^{2}}}$$

In this model, *Q* indicates the adequate heat power [W]; *Q* = *η*·*U*·*I*. H, *U*, and *I* denote the efficiency, welding voltage [V], and current [A], respectively; $f_{f}$ and $f_{r}$ represent the proportional coefficients at the front and rear ellipsoids of the heat source, respectively; $f_{f}$ + $f_{r}$ = 2. $a_{f}$, $a_{r}$, b, and c represent, respectively, the length/width/depth of the front/rear double-ellipsoidal heat source parameters, used to define the size and shape of the ellipses; and *x*, *y*, and *z* denote the local coordinates of the reconstructed models.

## 3. Results

### 3.1. Characterization of the Surface and Geometric Quality

#### 3.1.1. Quality and Printability of the Single-Wall Surface

**Figure 4:**
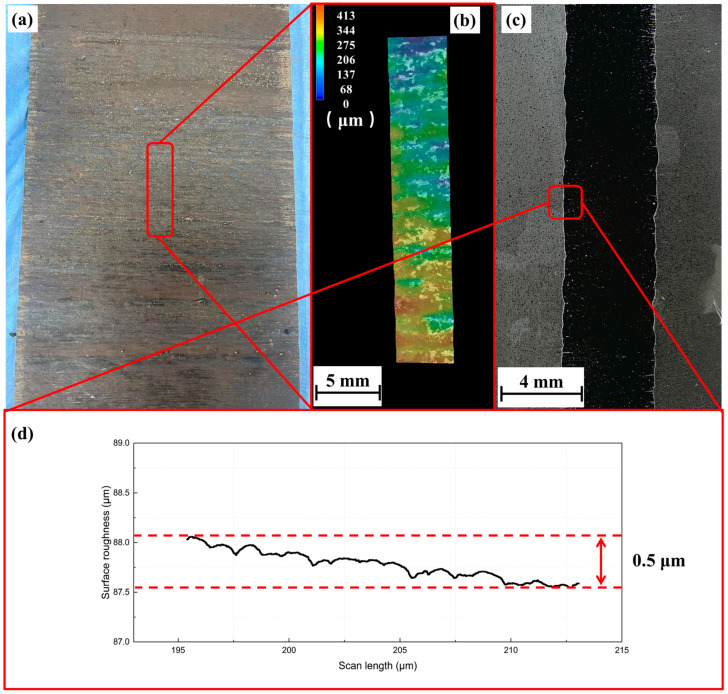
Surface investigation of the single-wall deposition of 17-4PH stainless steel. (**a**,**b**) specimen surface and its results; (**c**,**d**) cross-section results.

A single-wall sample test was conducted before the grid fin components were printed. In addition, several orthogonal experiments were performed using multiple parameters, and the melt pool’s morphology was assessed to allow for the selection of the optimal parameter set for single-wall printing. Figure 4a illustrates a printed sample. In this region, a Keyence digital microscope was used to obtain 3D surface images, revealing an approximate range of 0–413 μm. Figure 4c shows the transverse cross-section of a specimen, displaying the surface quality precisely. We examined surface fluctuations of approximately 0.5 μm within an 18 μm range, and profilometer measurements were carried out, which indicated an excellent surface quality, as shown in Figure 4d.

**Figure 5:**
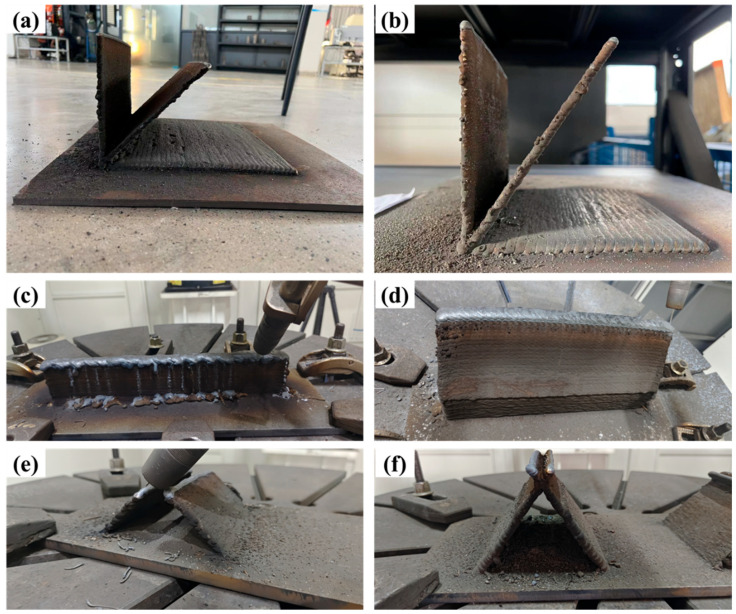
Tests for different tilt angles for S-mode deposition: (**a**,**c**,**e**) for 45 degrees and (**b**,**d**,**f**) for 55 degrees.

In previous studies, the L mode has been widely studied, with the results indicating its high quality based on single-wall samples in this mode. However, the opposite results have been reported for the S mode. Currently, the feasibility of the S mode remains poorly understood, especially at different printing angles. Therefore, we tested the feasibility of single-wall printing at various angles under the S mode. Figure 5a and b display unsupported 4 mm thick single-wall prints at 45 and 55 degrees, respectively, indicating stable printing under these conditions. Notably, when the wall thickness was increased to 20 mm, the 45-degree specimen was rendered inappropriate due to instability, collapse, and an incomplete surface. However, its quality unexpectedly improved from 45 to 55 degrees (Figure 5d). Based on these findings, a 55-degree inclination was deemed more suitable for single-wall printing under the parameters tested. The feasibility of cross-printing the intersection joints was also validated for the grid fin intersections, and the 45-degree and 55-degree intersections are presented in Figure 5e and f, respectively. The results demonstrated that the end node of the intersection was influenced by the collisions of the weld gun at 45 degrees due to the angle constraints, which increased the difficulty of completing the print. At 55 degrees, however, the deposition specimen seemed to achieve better results (Figure 5f). Thus, the 55-degree printing angle met the production requirements for grid fins by allowing for a suitable thickness and intersection joint.

#### 3.1.2. Evaluation of the Geometry of the Grid Fins

The geometric properties of the grid fins were measured using 3D scanning technology with the FreeScan UE Pro laser scanner (SHINING 3D, Hangzhou, China) at Zhejiang University. Point clouds for the grid fins were generated and analyzed using the SpatialAnalyzer (SA 2024.2) industrial geometry software at the University of Bath. In the absence of geometric and positioning constraints during scanning, all of the outer and inner surfaces of the grid fins were captured. Geometric deviations were processed using the best-fitting approach (see Table 3 for results).

**Figure 6:**
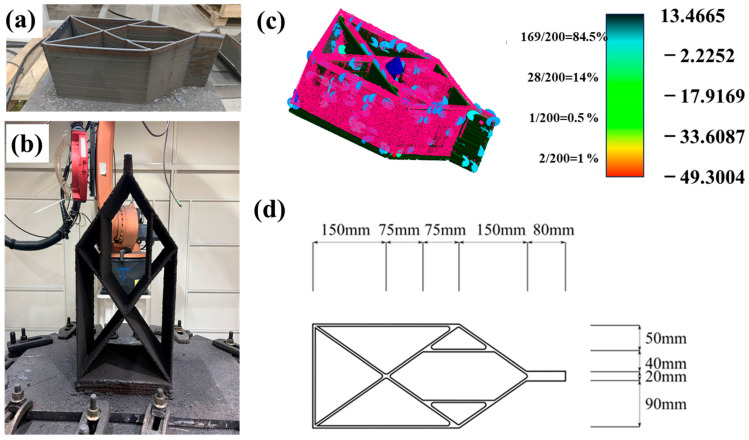
Typical geometry of grid fins in two printing modes: (**a**,**b**) experimental specimen for L and S mode and (**c**,**d**) geometry results and original structure.

Figure 6a and b show an L-mode specimen and an S-mode specimen, respectively. Figure 6c illustrates the alignment between the measured point clouds of the L model (in dark) and the S model (in pink), displaying the significant geometric deviations, with relatively larger deviations observed near the corner regions and regions with small deviations (in blue) dominating the entire pattern. Figure 6d illustrates the detailed dimensions of a specimen in the plane view at a nominal height of 200 mm. The results shown in Table 3 suggest that the geometric deviations between the physical specimens and the digital models were decreased following the elimination of the errors, with a maximum deviation of 5.26 mm. The S grid fin, given its orientation and exposure to greater external forces, may necessitate more stringent geometric control compared to the L grid fin, which exhibits lower overall deviations.

### 3.2. Microstructure and Mechanical Properties

#### 3.2.1. Microstructure of the L- and S-Mode Joints

**Figure 7:**
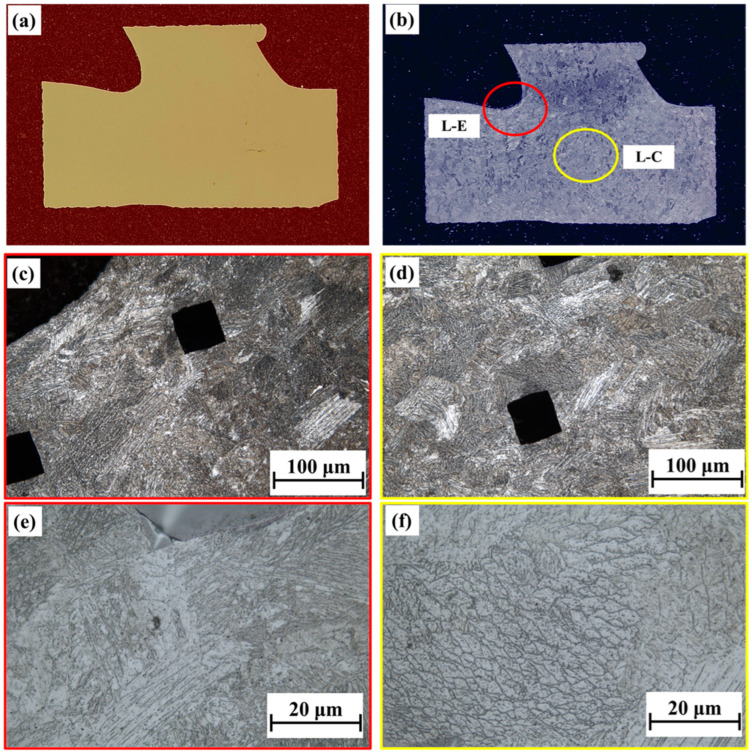
Illustration of the macrostructure and microstructure of the intersection joint in L mode: (**a**,**b**) macrostructure for L mode with polishing and etching and (**c**,**e**) microstructure for L-E in 50 and 500 magnifications; (**d**,**f**) microstructure for L-C in 50 and 500 magnifications.

**Figure 8:**
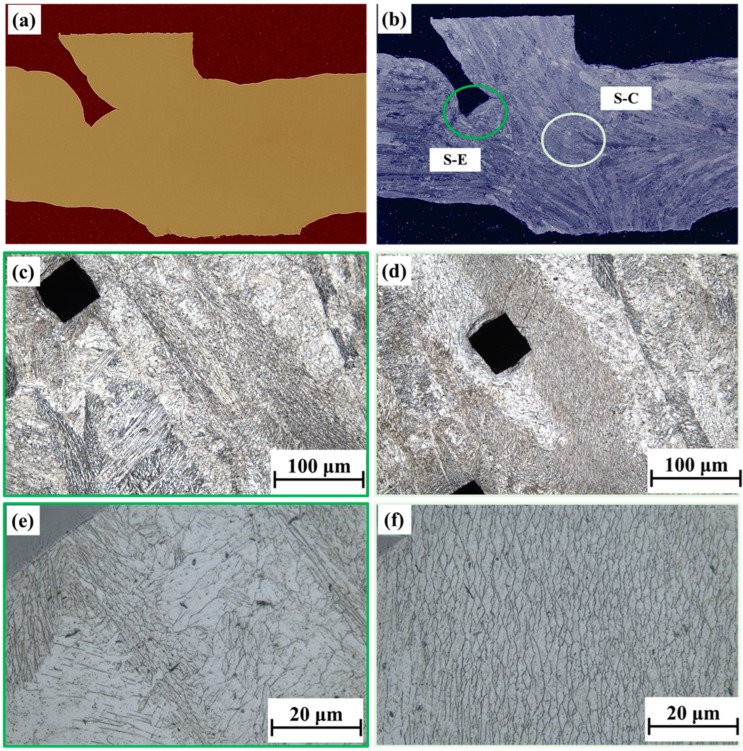
Illustration of the macrostructure and microstructure of the intersection joint in S mode. (**a**,**b**) macrostructure for S mode with polishing and etching and (**c**,**e**) microstructure for S-E in 50 and 500 magnifications; (**d**,**f**) microstructure for S-C in 50 and 500 magnifications.

Figure 7 shows the microstructure under L-mode deposition, both for polished and etched surfaces. The polished surface appeared to be a smooth surface with no apparent porosity and a lack of fusion defects. After etching, the edge and central regions of the node were examined under varying magnifications. The red circle indicates the edge (L-E) in Figure 7b, whereas the yellow circle indicates the central area (L-C). Since the microstructure is a cross-section of the building direction, the grains are not shown as columnar grains but equiaxed grains. In the enlarged region, the structure is visualized in great detail. The results demonstrate no significant differences between the microstructures in the edge and center regions, with both composed of martensite and a small amount of δ-ferrite retained (dendrite residual between two martensite regions). Notably, the S mode differs from the L mode. In Figure 8b, S-E and S-C represent the edge and internal microstructures, marked with green and yellow-green circles, respectively. Specifically, the S mode presents a higher number of columnar grains owing to the interface in the longitudinal direction of deposition. Although there were no significant inclusions or cracks in the polished state, no fusion and sharp-angle cracks were seen in the S-E region. Under the complex environment, these defects caused stress concentration, increasing the risk of crack propagation. The further microstructural analysis performed using an SEM and EBSD is presented in the subsequent sections.

#### 3.2.2. Hardness of the Various Cross-Sections of the Joints

**Figure 9:**
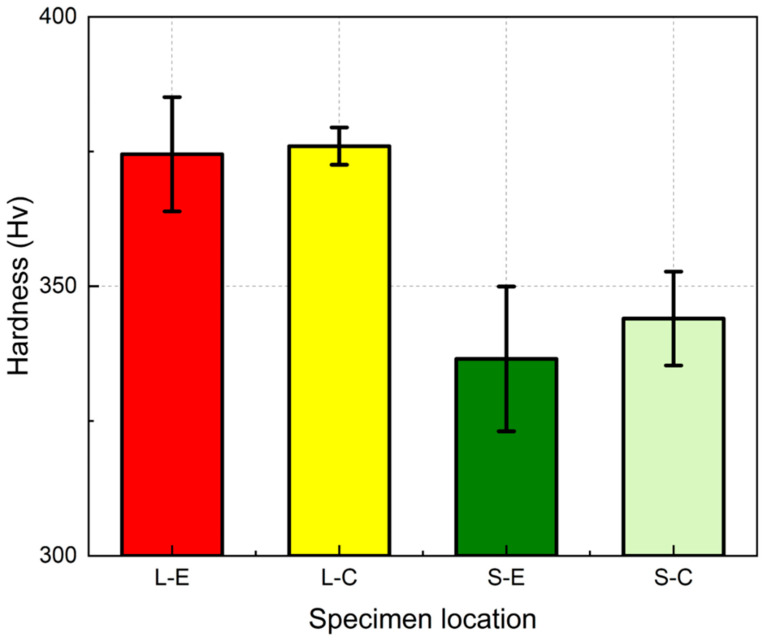
Hardness of different regions in L and S modes.

The results indicated that the hardness varied significantly between the two printing modes. In Figure 9, red and yellow represent the hardness values at the edge (L-E) and center (L-C) under the L mode, respectively, while green and light green represent the corresponding areas in the S mode. The average hardness under the L mode is approximately 375 Hv, which is significantly higher compared with that of the S mode (around 340 Hv) across both the edge and center regions. Moreover, the difference in the hardness between the edge and center is minimal under each mode.

#### 3.2.3. Mechanical Properties of Various Cross-Sections of the Joints

**Figure 10:**
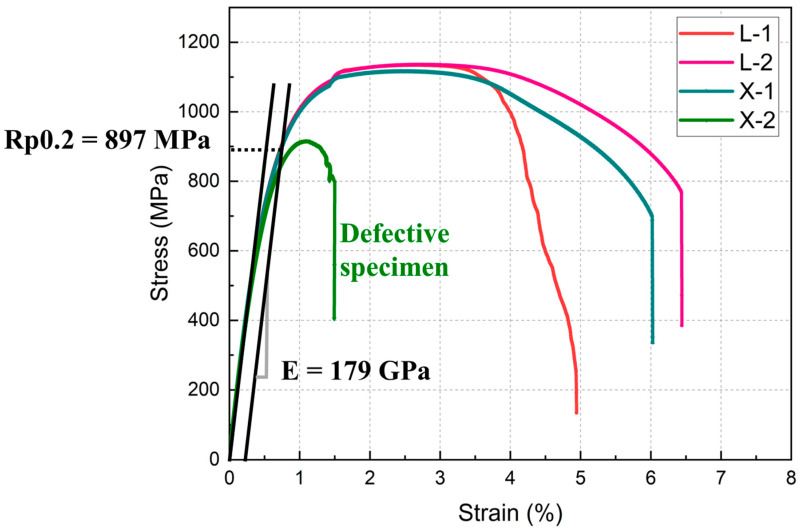
The mechanical properties of different specimens in L and S modes.

The mechanical properties of the joints are presented in Figure 10. Of note, the two tensile samples were recorded under both L mode and S mode, denoted as L-1, L-2, S-1, and S-2, and are differentiated by color. The mechanical properties of the samples fabricated in L mode displayed a high degree of uniformity. Minimal discrepancies were observed in the tensile and yield strength, and the elongation exhibited a variation of only 1.4%. However, significant variations were observed in the mechanical properties at the joints of the samples printed under the S mode, especially in S-2, where the elongation was 1.5%, markedly below the average. This is likely attributed to the lack of fusion and sharp-angle cracks in the S mode, inducing premature fracture (Figure 8b). The strength of the S mode samples was approximately 20 MPa lower than that of the L mode samples, which was consistent with results of the hardness tests.

## 4. Discussion

The L and S modes each exhibit distinct advantages in practical applications. From industrial and economic perspectives, the S mode has potential in terms of its performance compared to the L mode. However, due to its stable properties, the L mode may be more suited to industrial applications. This study demonstrates some critical differences between the L and S printing modes: (1) The L mode improves the stability of the surface quality, making it a more straightforward scanning strategy and achieving a higher overall print quality. (2) In S printing, the base size is reduced and the fixture requirements are lowered for ultra-large samples, significantly improving its feasibility for large-scale products. Therefore, a comprehensive analysis of the variations in the microstructure and performance between the two deposition methods is advocated. Temperature distribution simulation results can be leveraged to clarify the association between the microstructure and the mechanical properties. Moreover, it is crucial to calculate and evaluate the deformation and residual stress in the L mode and the S mode to enhance the selection for large components.

### 4.1. The Effect of the L and S Modes on the Microstructure and Mechanical Properties

**Figure 11:**
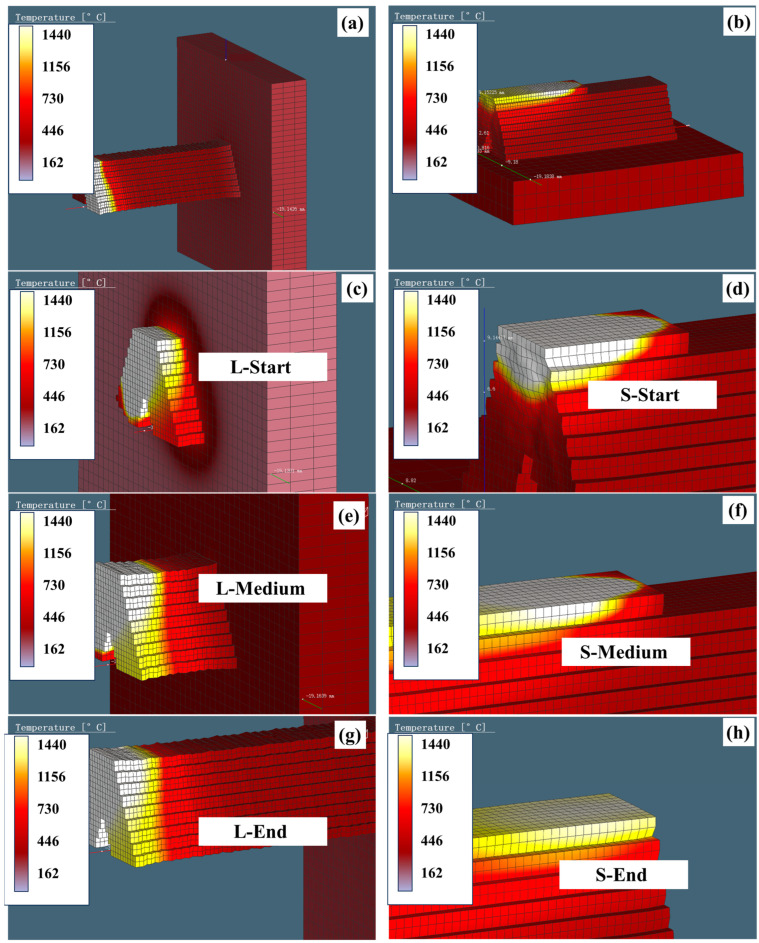
Thermal history simulation results at different times: (**a**,**c**,**e**,**g**) for L mode and (**b**,**d**,**f**,**h**) for S mode.

The thermal histories under the two modes (L and S) are shown in Figure 11a and b, respectively. The simulated melt pool characteristics, including its morphology, temperature distribution, and peak temperatures, appear to be within reasonable limits. The thermal histories at key nodes were extracted at different time intervals to explore the microstructural evolution under each mode. Subsequently, they were labeled as start, medium, and end.

**Figure 12:**
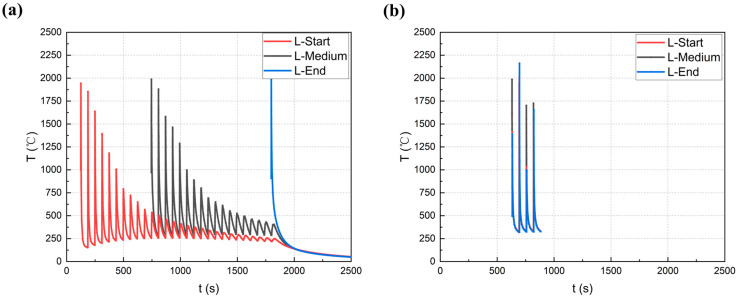
Simulation of thermal cycles for L (**a**) and S (**b**) modes in different regions.

The extracted thermal history curves for the L and S modes are shown in Figure 12a and b, respectively. In the L mode, the thermal history at the node varies, which is influenced by the additive manufacturing (AM) layers. In the initial layer, located at the base of the deposition, there were significantly more thermal cycles than in subsequent deposition layers. This phenomenon is most clearly observable in the middle and final portions of the build. It is important to emphasize that the additive manufacturing process concludes with a single thermal cycle. Different thermal histories affect the hardness and mechanical properties, inducing variations in microstructure. Consequently, the mechanical properties in the L mode my exhibit inhomogeneous properties. Notably, there were fewer thermal cycles at the intersection joint under the S mode because the cross-sectional deposition region was unaffected by the initial welding thermal history. Due to the consistent thermal history experienced during S-mode deposition, the resulting microstructure exhibits greater homogeneity compared to that under the L-mode. This is attributed to the minimal variation in the thermal history throughout the welding process in the S mode. In contrast, the L mode, with its more dynamic thermal cycles, leads to a more heterogeneous microstructure. However, both modes are susceptible to defects such as fusion (LOF). This highlights the significance and motivation for further research into the S mode, particularly from the perspective of quality optimization.

**Figure 13:**
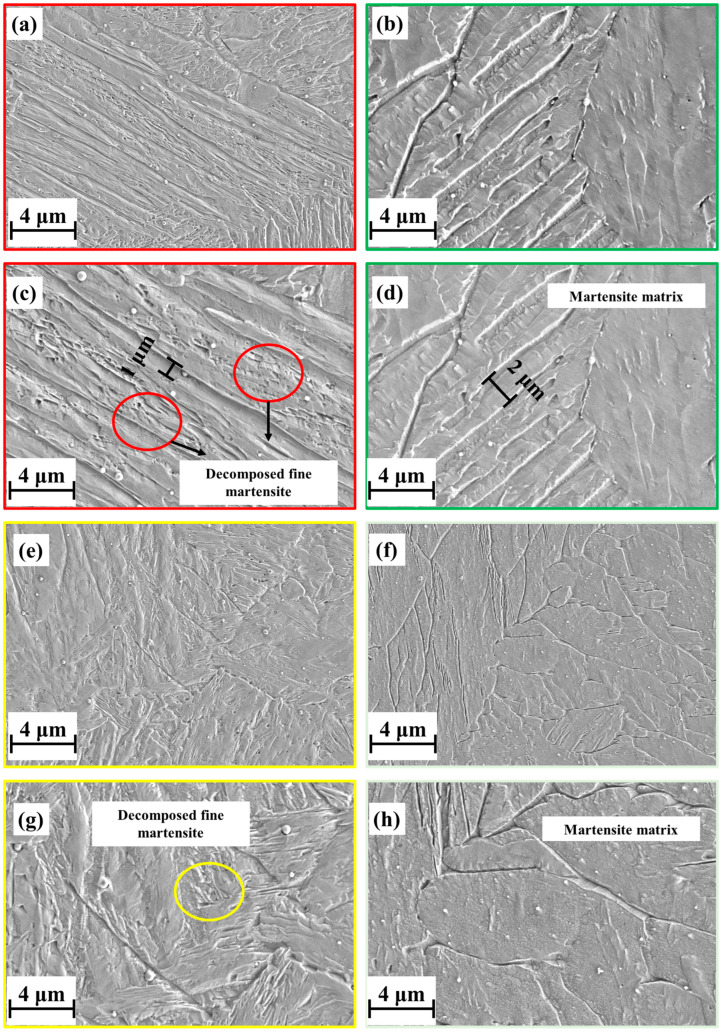
Microstructure under high magnification for the L deposition mode (**a**,**c**,**e**,**g**) and the S deposition mode (**b**,**d**,**f**,**h**).

To facilitate a more in-depth structural analysis, these areas were examined further using an SEM. Figure 13a,c depict the microstructure at the edge observed in the L mode, while Figure 13b,d illustrate the corresponding microstructures in the S mode. Despite the lath martensite exhibited in both structures, the thickness was still different between the structures. The martensite was finer in the L mode, at approximately 1 μm, indicating a reduction of at least 50% relative to the S mode. Additionally, the martensite contained more decomposed fine martensite structures in the L mode. Similarly, the grain refinement varied significantly in the central region (Figure 13e–h). Notably, the martensite formed in the L mode exhibited finer refinement than that in the S mode for both lath and block martensite. The decomposed, fine martensite was pronounced in the martensite matrix. Regardless of the location (edge or center), the volume fraction of fine martensite was consistently higher in the L mode compared to the S mode. This disparity can be attributed to the differential thermal cycling experienced by the materials. An increase in the thermal cycles promotes the refinement of the martensite within the martensitic matrix.

**Figure 14:**
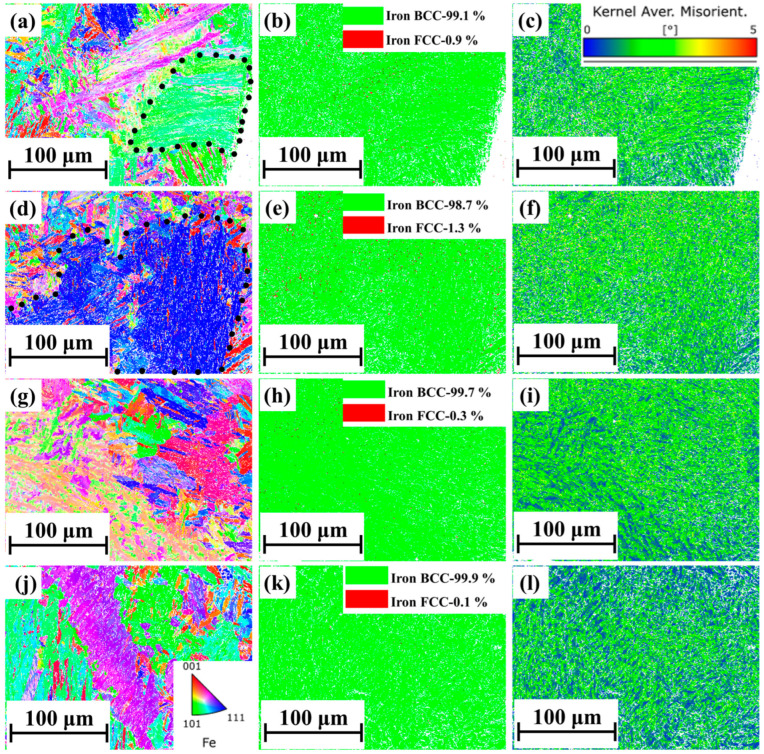
EBSD-generated maps; (**a**,**d**,**g**,**j**) IPF map for L-E, L-C, S-E and S-C; (**b**,**e**,**h**,**k**) Phase map for L-E, L-C, S-E and S-C; (**c**,**f**,**i**,**l**) KAM map for L-E, L-C, S-E and S-C.

Figure 14 displays the EBSD images of the joint regions under the L and S modes. Figure 14a, Figure 14d, Figure 14g, and Figure 14j show the original IPF maps for L-E, L-C, S-E, and S-C, respectively. The IPF maps are distinct from the standard random orientation of martensite in the S mode, while the L mode exhibits a colony-structure martensite with parallel orientations, which is highlighted by the black dashed lines. The second column (Figure 14b,e,h,k) presents the phase diagrams, in which green indicates the Fe-BCC phase (martensite or ferrite) and red represents the Fe-FCC phase (austenite). Notably, the phase proportions varied under different scanning strategies. The L mode contained at least three times more residual austenite than that in the S mode, where residual austenite was hardly identifiable. This observation is consistent with findings by Lyu et al. [6], where the proportion of residual austenite was approximately 1%. The third column presents KAM maps of different regions in the L and S modes. Both modes achieved similar results in the KAM maps, with no significant plastic strain observed.

### 4.2. The Effect of the L and S Modes on Displacement and Residual Stress

**Figure 15:**
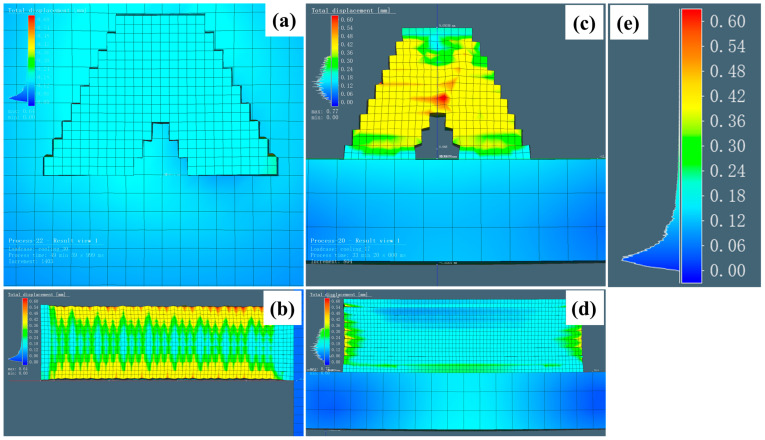
Simulation of displacement with different perspectives in the L mode (**a**,**b**) and the S mode (**c**,**d**). Enlarged scale bar value is shown in (**e**).

Figure 15 shows the displacement results obtained under the L and S modes. To accurately evaluate the displacement under the two printing modes, a similar geometry and mesh of the components were used, differing only in the printing mode. The total displacement color ranged from 0 and 0.6 mm, with the same end time. Figure 15a,b illustrate the simulation results obtained using the L mode model from two different perspectives. The S mode is shown in Figure 15c,d. The displacement color scale bar is presented in Figure 15e to provide better visualization. In the intersection region, the displacement of the two modes varied significantly. The displacement in the L mode was minimal, ranging from approximately 0.1 to 0.2 mm. In contrast, the S mode exhibited pronounced yellow regions in the cross-section, with the corresponding displacement observed in the range of 0.3–0.4 mm. In the central region, the maximum displacement approached 0.6 mm. This disparity arose from the more concentrated temperature distribution at the intersection joint in the S mode, leading to an accelerated cooling rate. Furthermore, the experimentally observed poor surface quality in the S mode contributed to higher displacement. Figure 15b,d provide side views of the model for visual reference. In the L mode, the displacement was concentrated on the upper surface, as determined following the deposition trajectory. Regions with pronounced displacements were mainly concentrated at the fusion interlayers, the start or end points of the arcs, and the component surface. In contrast, the S mode was relatively stable. Due to the shorter deposition trajectory, the thermal distribution was reduced, causing the minimum displacement.

**Figure 16:**
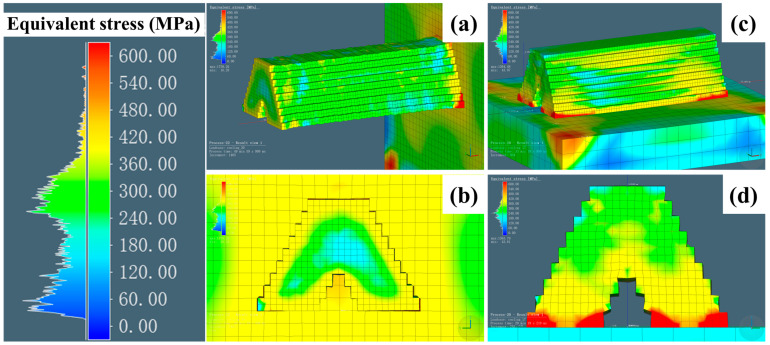
Simulation of residual stress with different perspectives in the L mode (**a**,**b**) and the S mode (**c**,**d**).

**Figure 17:**
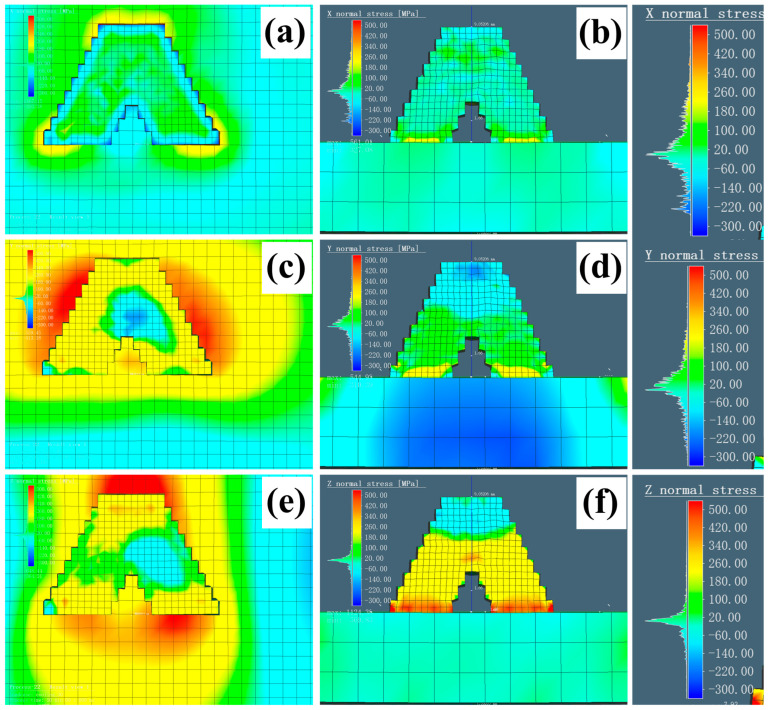
Simulation of residual stress in each direction with different perspectives in the L mode (**a**,**c**,**e**) and the S mode (**b**,**d**,**f**).

Besides displacement, residual stress can significantly affect the mechanical properties. Therefore, it is important to evaluate the weak points to optimize the structures and processes. The equivalent stress (Von Mises Stress) ranging from 0 to 600 MPa is indicated by different colors. Figure 16a,b illustrate the L mode, while Figure 16c,d show the S mode. Similar to the displacement analysis, the equivalent stress distribution exhibited significant variations when viewed from two different perspectives. The regions with the highest residual stress were those where the components came into contact with the substrate. This phenomenon is common to both printing modes and occurs in the initial layer. Notably, the location of this phenomenon varies depending on the printing strategy. In Figure 16a (L mode), it is situated on the right, while in Figure 16c (S mode), it is located at the bottom. When the component was removed from the substrate, the regions with an equivalent stress near 600 MPa were eliminated. The range of the maximum stress regions in yellow is roughly from 360 to 420 MPa. In comparison with the yellow areas, the L mode exhibited a stress concentration only at the surface, with stress below 180 MPa at the intersection joints (blue region). At the end of the surface machining, the component’s stress become homogenous (green region). In contrast, yellow regions were observed in the central intersection area under the S mode, with an equivalent residual stress of approximately 420 MPa, exceeding the highest 180 MPa recorded in the L mode. Moreover, the yellow areas extended over most of the side surfaces. That is, the residual stress under the S mode remained significantly higher compared with that of the components printed in the L mode and was randomly distributed, even after secondary surface machining. Although the simplified structure did not fully represent the integrity of actual grid fins, it allowed for the prediction of the trends in the residual stress and provided insights for post-heat treatment when the S mode is employed. The residual stress in the other directions (X, Y, and Z) is presented in Figure 17, laying the foundation for future predictions of its negative impact on fatigue life.

## 5. Conclusions

Based on the present results, the following conclusions are drawn regarding the effect of the deposition strategy (L and S) for grid fin components on the microstructure and mechanical properties of wire-arc-additive-manufactured 17-4PH stainless steel.

1. Among the arc additive manufacturing methods for grid fins, the L mode process is simple and more stable than the S mode process. This is especially true at a certain inclination angle, as the overlap fusion is better in the horizontal mode, which allows for greater angle flexibility (both 45 and 55 are usable) of the crossover structures. The surface quality under the L mode is also better than that under the S mode.
2. Due to the microstructural differences caused by the printing methods, the perspectives in terms of the grain observations are distinct. The L mode yields equiaxed grains, while the S mode produces more columnar grains. The L mode, characterized by a higher number of thermal cycles, leads to the formation of decomposed fine martensite. This results in a superior fusion quality at the intersection joint compared to the S mode. However, the absence of defects in the L mode can potentially compromise the stability of the mechanical properties and pose significant safety risks.
3. The simulation results revealed a significant difference in the displacement and residual stress between the L and S modes. When the grid fin was printed under the L mode, the residual stress was concentrated at the bottom, indicating a decreasing trend with subsequent deposition. In contrast, the stress persisted with no decrease observed under the S mode as the height increased. Instead of spreading to the surface like under the L mode, the maximum displacement and residual stress accumulated increasingly in the center under the S mode.

## Figures and Tables

**Table 1:** The chemical composition of the 17-4PH welding wire.

Element	C	Mn	Si	Cr	Ni	Mo	Cu	Nb	Fe
wt (%)	0.0092	0.502	0.426	16.4	4.52	0.007	3.39	0.227	Bal.

**Table 2:** The processing parameters of the WAAM experiment.

Welding Condition	Values
CMT current (A)	117
CMT voltage (V)	19
Welding speed (mm/s)	4.3
Feed rate (m/min)	6
Gas flow rate (L/min)	20

**Table 3:** Geometric deviations between standing and flat grid fins.

Category	After Eliminating Errors		Without Eliminating Errors	
	Standard	Absolute	Standard	Absolute
Average	0.04	1.41	−0.80	3.32
Max	5.26	5.26	13.47	49.3
Min	−4.98	0.05	−49.3	0.05
Std.	1.22	1.23	5.87	5.94

## Data Availability

The raw data supporting the conclusions of this article will be made available by the authors on request the rocket company secrets.
